# Supplementary figures and images for: NXP031 prevents dopaminergic neuronal loss and oxidative damage in the AAV-WT-α-synuclein mouse model of Parkinson’s disease
Source: PLoS One. 2022 Jul 28;17(7):e0272085. doi: 10.1371/journal.pone.0272085 (PMC9333296; doi:10.1371/journal.pone.0272085)

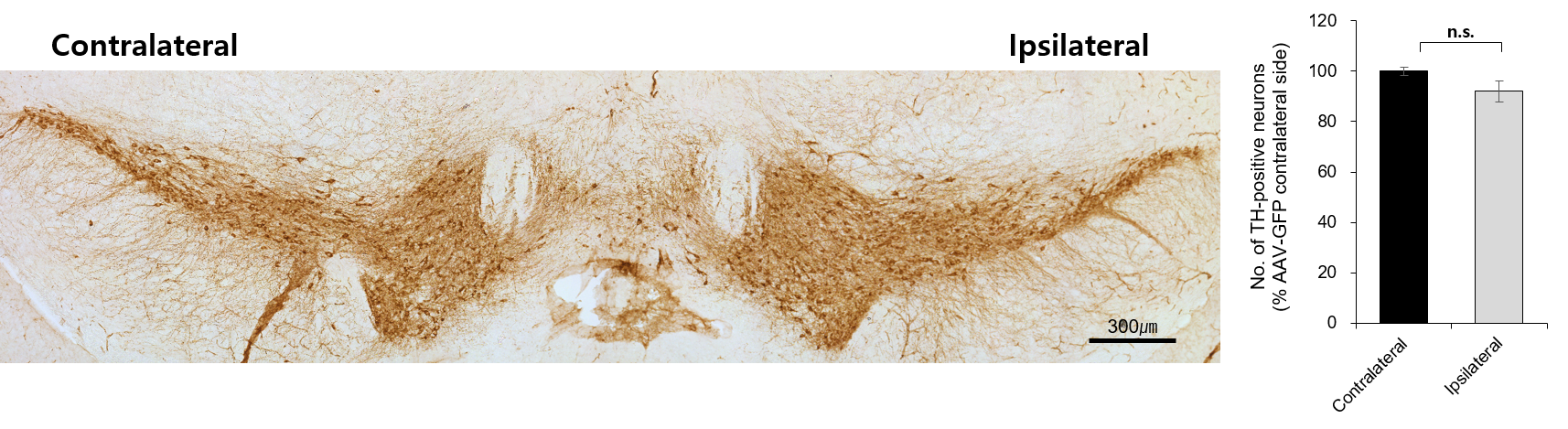

Supplement: S1 Fig — Representative micrographs of TH immunostaining in the SN. (TIF) [file pone.0272085.s001.tif]

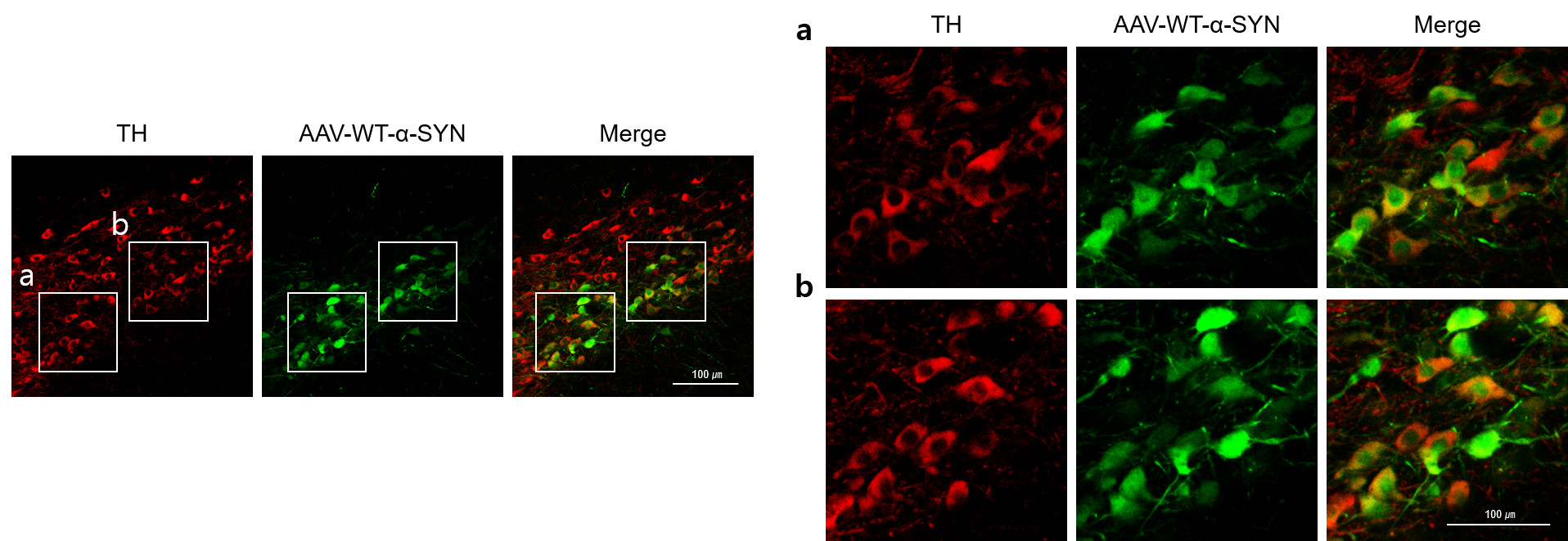

Supplement: S2 Fig — (A) Representative micrographs of TH immunostaining in the SN. (B) % of TH-positive neurons in the SN compared to the contralateral side. Data are presented as the mean ± S.E.M. (Student t-test, n = 6 mice). (TIF) [file pone.0272085.s002.tif]
