# Supplementary material for: NXP031 prevents dopaminergic neuronal loss and oxidative damage in the AAV-WT-α-synuclein mouse model of Parkinson’s disease
Source: PLoS One. 2022 Jul 28;17(7):e0272085. doi: 10.1371/journal.pone.0272085 (PMC9333296; doi:10.1371/journal.pone.0272085)
Supplement: Attachment — Submitted filename: Response to reviewers - Final.docx [file pone.0272085.s003.docx]

Hemant K. Paudel

Academic Editor

PLOS ONE

We appreciate the constructive comments made by reviewers and giving us the opportunity for revision. We have revisited the entire manuscript and thoroughly revised the manuscript accordingly, and we are confident that our manuscript satisfies reviewers’ concerns and is greatly improved.

Prior to point-by-point answers to each question raised by reviewers, we would like to clarify the AAV-WT-α-Syn PD model that we used in this study. We noticed common concerns regarding hippocampal vs. nigral pathology and related issues. The primary aim of using this model is to test if NXP031 effectively reduces or blocks α-Syn aggregation-induced dopaminergic (DA) neuronal loss in the SN (not hippocampal dysfunction or loss). To assess the therapeutic efficacy, we measured 1) the number of TH-positive neurons in the SN, 2) pS129-α-Syn as a marker for α-Syn aggregation, and 3) 4-HNE as oxidative lipid peroxidation, and 4) γH2A.X as a DNA double-strand break. DA neuronal loss in the saline ingested group was significantly obvious compared to the NXP031 treated group. This evident DA neuronal loss invited difficulty in measuring other pathology markers (2-4) as all markers express inside cells and disappear with degeneration. Therefore, we investigate the hippocampus as an alternative region for two reasons: 1) propagation of α-Syn aggregates from the SN to the hippocampus over time is well documented (ref below), 2) hippocampal neuronal loss is minimal even though α-Syn pathology is observed (shown in our nissl staining in the hippocampus in Fig. 5). In the hippocampus, we were able to measure changes in pathologic markers between groups, confirming NXP031 blocked lipid peroxidation, α-Syn aggregation, and DNA damage. As these pathologic markers were also similarly detected in some remaining neurons in the SN (Figure below), what we observed in the hippocampus could indicate the neuroprotective mechanisms underlying how NXP031 prevents α-Syn-mediated DA neuronal degeneration.

**Images of TH, pS129-α-Syn, and 4-HNE immunostaining in the same mouse brain tissues of the AAV-WT-α-Syn group.** **a.** The periphery of AAV-WT-a-Syn injected site. Compared to the core region (**b**), this region received relatively mild damage, and the neuronal cell bodies still remained, allowing that immunostaining for pathologic markers was observed. In addition, we could see stronger pS129-α-Syn and 4-HNE immunostaining than NXP031 treated group. **b.** The core of AAV-WT-a-Syn injected site. Severe damage caused by overexpression of AAV-WT-a-Syn led to almost complete loss of dopaminergic neuronal cell bodies. TH, pS129-α-Syn, and 4-HNE immunostaining could not be detected

Kim, S., Kwon, S. H., Kam, T. I., Panicker, N., Karuppagounder, S. S., Lee, S., ... & Ko, H. S. (2019). Transneuronal propagation of pathologic α-synuclein from the gut to the brain models Parkinson’s disease. *Neuron*, 103(4), 627-641.

**Reviewer#1**. In the current manuscript, Kim et al. describe the effects of novel vitamin C derivative, NXP-031, on a Parkinson's disease mouse model based on AAV-alpha-synuclein injection on substantia nigra. Although the results are interesting, particularly the protective effect of NXP-031 on TH+ neuron count, there are several issues that needs to be addressed.

**Q1**. My main concern is that the authors do not show any data from the striatum, where aSyn injected in substantia nigra is eventually transported? That is anyway the equally important area (or in early disease even more important) as substantia nigra in Parkinson’s disease pathology, and most of the endogenous aSyn is located in presynaptic area. I do understand hippocampus as one of the projection areas of substantia nigra where aSyn is also seen but why the authors did not study striatum and e.g. motor cortex that are more important for locomotor deficits. Moreover, as the authors use unilateral model of AAV-aSyn, a model measuring unilateral deficits e.g. cylinder test or staircase test would be probably more informative than rotarod assay, and gives usually more information on striatal damages than rotarod assay.

**A1**. We genuinely appreciate all comments of reviewer #1.

PD model using AAV-mediated intra-nigral overexpression of α-synuclein (α-Syn) mainly targets dopaminergic (DA) neurons in the substantia nigra (SN), causing their degeneration via toxic aggregation of α-Syn over time. As DA neurons in the SN undergo degeneration, striatal DA neuronal processes are also degenerated. As reviewer suggested, we performed IHC for TH in the striatum and found striatal TH immunostaining density was accordingly protected by NXP301. This new data is included in this revised manuscript (Fig 2A and B).

In α-Syn/AAV-mediated unilateral PD model, as reviewer suggested, behavioral deficits are assessed using the tools measuring motor asymmetries, such as amphetamine or apomorphine-induced rotations, cylinder test, and paw stepping test. Unfortunately, we did not include these tests in the current study. Instead, we used the rotarod test which has been widely used and well appreciated to reliably assess motor behavioral changes in the unilateral PD models [1-4].

[1] An, H., Lee, H., Yang, S., Won, W., Lee, C. J., & Nam, M. H. (2021). Adenovirus-induced Reactive Astrogliosis Exacerbates the Pathology of Parkinson’s Disease. *Experimental Neurobiology*, 30(3), 222.

[2] Becker, G., Michel, A., Bahri, M. A., Mairet-Coello, G., Lemaire, C., Deprez, T., ... & Plenevaux, A. (2021). Monitoring of a progressive functional dopaminergic deficit in the A53T-AAV synuclein rats by combining 6-[18F] fluoro-Lm-tyrosine imaging and motor performances analysis. *Neurobiology of aging*, *107*, 142-152.

[3] Bu, L. L., Liu, Y. Q., Shen, Y., Fan, Y., Yu, W. B., Jiang, D. L., ... & Wang, J. (2021). Neuroprotection of exendin-4 by enhanced autophagy in a parkinsonian rat model of α-synucleinopathy. *Neurotherapeutics*, *18*(2), 962-978.

[4] Oliveras-Salvá, M., Van der Perren, A., Casadei, N., Stroobants, S., Nuber, S., D’Hooge, R., ... & Baekelandt, V. (2013). rAAV2/7 vector-mediated overexpression of alpha-synuclein in mouse substantia nigra induces protein aggregation and progressive dose-dependent neurodegeneration. *Molecular neurodegeneration*, 8(1), 1-14.

**Q2**. Additionally, the authors do not provide any information about the aSyn aggregation itself, particularly when the pS129 aSyn did not show any reliable staining in the substantia nigra. If they consider that the mechanism-of-action for NXP031 is antioxidant mechanism, they should also show some antioxidant response markers in the substantia nigra, e.g. SOD1, catalase.

**A2-1**. We used phosphorylated S129-α-Syn, which is associated with the facilitation of LB formation and accelerated neurodegeneration in PD, as a marker for α-Syn aggregation [1-3]. A previous study testing various commercially available monoclonal antibodies for reliable detection of pS129-α-Syn inclusions confirmed that the pS129-α-Syn antibody (EP1536Y, Abcam) used in this study exhibited the highest sensitivity and specificity [3]. As the reviewer noticed, we could not see a clear pS129-α-Syn signal in the SN. It is due to evident DA neuronal loss at the point we investigated. As pS129-α-Syn is expressed inside DA neurons, it is hard to see its signal after DA neuronal degeneration. We also changed and added the representative images (Fig 3A and B) to show more convincing pS129-α-Syn staining in the SN. We also performed double immunostaining of pS129-α-Syn and TH to show that DA neurons mainly express pS129-α-Syn in the SN where α-Syn/AAV injected.

Fig 3.

[1] Arawaka, S., Sato, H., Sasaki, A., Koyama, S., & Kato, T. (2017). Mechanisms underlying extensive Ser129-phosphorylation in α-synuclein aggregates. *Acta neuropathologica communications*, *5*(1), 1-15.

[2] Ghosh, D., Mehra, S., Sahay, S., Singh, P. K., & Maji, S. K. (2017). α-synuclein aggregation and its modulation. International journal of biological macromolecules, 100, 37-54.

[3] Gorbatyuk, O. S., Li, S., Sullivan, L. F., Chen, W., Kondrikova, G., Manfredsson, F. P., ... & Muzyczka, N. (2008). The phosphorylation state of Ser-129 in human α-synuclein determines neurodegeneration in a rat model of Parkinson disease. *Proceedings of the National Academy of Sciences*, *105*(2), 763-768.

[4] Delic, V., Chandra, S., Abdelmotilib, H., Maltbie, T., Wang, S., Kem, D., ... & West, A. B. (2018). Sensitivity and specificity of Phospho‐Ser129 α‐Synuclein monoclonal antibodies. Journal of Comparative Neurology, 526(12), 1978-1990.

**A2-2.** Ascorbic acid is a well-known antioxidant, especially in blocking lipid peroxidation (ref below). For NXP031 as an enhancer for vitamin C, therefore, we directly measured 4-HNE levels as a biomarker of oxidative stress, confirming NXP031 reduced lipid peroxidation.

Huang, H. Y., Appel, L. J., Croft, K. D., Miller III, E. R., Mori, T. A., & Puddey, I. B. (2002). Effects of vitamin C and vitamin E on in vivo lipid peroxidation: results of a randomized controlled trial. *The American journal of clinical nutrition*, *76*(3), 549-555.

**Q3**. The authors should also revise the manuscript more carefully, now there are several mistakes that make the interpretation of the results difficult: Figure 3, there are no details in the materials and methods about the pS129 aSyn analysis and that should be added. How many sections and brains were counted etc. The staining figures of pS129 aSyn also look puzzling. Why there is high amount of staining in the substantia nigra pars reticulata? I have some doubts about the antibody specificity, it appears to detect either normal aSyn as well or then some other unspecific protein. Moreover, Figure 5 should present the immunostaining of phosphorylated H2A in the hippocampus but it appears to present a Nissl staining? This needs to be corrected.

**A3-1.** We have added how to quantify the results of pS129-α-Syn, 4-HNE, and γH2A.X immunostaining.

Immunohistochemistry and immunofluorescence

To quantify pS129-α-Syn, 4-HNE, and pH2A.X staining in the hippocampus, pS129-α-Syn, 4-HNE, and γH2A.X positive cells were counted in 2 sections per mouse. Cell counting was performed blind to the identity of groups by two researchers who did not participate in this experiment. The two researchers' average values were taken as the representative values.

**A3-2**. Our pS129-α-Syn staining was clear, and a non-specific staining pattern was not observed. Here, we provide an image of pS129-α-Syn staining the entire SN, demonstrating specific expression. pS129-α-Syn was detected in the SN pars compacta, but not in the pars reticulata.

**A3-3**. In Figure 5, we performed double staining for γH2A.X and Nissl. The dark brown color is γH2A.X immunostaining, and the blue color is Nissl staining.

Other comments:

Introduction:

**Q4**. Page 3: “α-Syn aggregates spread from the gut via the vagus nerve or anterior olfactory nucleus to the brain as the disease progresses [3].” This is indeed seen in the experimental animals and certain support in clinical disease comes from Braak staging (2003) but I would still avoid such strong statements on this as aSyn may well start aggregating in brain neuronal cells without propagating species.

**A4**. According to the reviewer’s suggestion, we have revised the sentence.

Introduction

Parkinson's disease (PD) is a common neurodegenerative disease. The neuropathological hallmarks of PD consist of the progressive loss of dopaminergic neurons in the substantia nigra (SN) and inclusions of aggregated α-synuclein (α-Syn), called Lewy bodies and Lewy neurites, in neurons [1, 2]. The Braak hypothesis postulates that α-Syn pathology can spread from the gut via the vagus nerve or anterior olfactory nucleus to the brain as the disease progresses [3]. α-Syn aggregates spread widely to the various interconnected brain regions by cell-to-cell propagation [4].

**Q5**. Page 3.” Additionally, vitamin C concentrations remain high in the central nervous system [15].” In the reference 15 (Nualart et al.) there were no neuronal cells used, and the publication is completely performed by using cell cultures and primary cells. How the authors can claim based on this reference that vitamin C concentrations remain high in the CNS?

**A5**. According to the reviewer’s advice, we have replaced the reference with new ones [1-3], showing thatascorbic acid concentrations were maintained high in the central nervous system compared to other peripheral systems.. In addition, other references showed that the concentrations of ascorbic acid in neurons and glia of the rat brain tissue were maintained high.

[1] Spector, R. (1977). Vitamin homeostasis in the central nervous system. *New England Journal of Medicine*, *296*(24), 1393-1398.

[2] Rice, M. E., & Russo-Menna, I. (1997). Differential compartmentalization of brain ascorbate and glutathione between neurons and glia. *Neuroscience*, *82*(4), 1213-1223.

[3] Rice, M. E. (2000). Ascorbate regulation and its neuroprotective role in the brain. *Trends in neurosciences*, *23*(5), 209-216.

**Q6**. Page 3. Several studies have shown that dietary supplements of vitamin C are not protective for PD, and authors should be careful not to draw conclusions about the beneficial effects of vitamin C on PD based on one study that has also raised some concerns (Tomoyuki Kawada; Reader Response: Dietary Antioxidants and the Risk of Parkinson Disease: The Swedish National March Cohort, 2021).

**A6**. Thank you for providing these insights. We are fully aware of the inconsistent results of previous studies on the effects of vitamin C on PD. In consideration of this part, we have revised the introduction section.

Introduction

Ascorbic acid (vitamin C) is a natural water-soluble vitamin and plays an essential role as a powerful antioxidant against free radicals [10]. Disappointingly, many studies have reported that vitamin C intake from diet and supplements had no effect on mitigating the risk of PD [11-13]. However, recently the Swedish National March Cohort long-term longitudinal study reported the positive effects of dietary vitamin C intake on PD risk. Participants in the highest tertile of dietary vitamin C showed reduced PD risk compared with those in the lowest tertile [14]. A recent study to confirm the effect of vitamin C in the MTPT-induced PD model demonstrated that vitamin C reduced dopaminergic neuronal loss by alleviating neuroinflammation such as microglial responses and astrocyte activation [42]. Additionally, vitamin C concentrations remain high in the central nervous system [15], and vitamin C has shown neuroprotective effects in vitro [16, 17]. Although there is still debate about the link between vitamin C and PD, it is clear that vitamin C is one of the excellent candidates for treating PD. Despite this, there are significant hurdles in developing Vitamin C-based therapeutic agents for neurodegenerative diseases. Vitamin C is rapidly oxidized and loses its antioxidant activity in the body, and it does not freely cross the blood-brain barrier.

Materials and methods:

**Q7**. Page 4-5: Why there is no AAV-empty + NXP031 group? AAV-GFP can also be toxic for the TH+ neurons (Albert et al. J Neurosci Res 2019), was this seen in the GFP-group?

**A7-1**. We did not include the AAV-GFP + NXP031 group because, as expected, the initial TH staining of this group did not show a difference from the AAV-GFP group. We did not include this group in the original manuscript to reduce time and effort. According to the reviewer’s advice, we included the AAV-GFP + NXP031 group in the revised manuscript. We rewrote the methods and the results sections accordingly.

**A7-2.** It has been shown that a high titer of AAV causes neurotoxicity (or DA degeneration when injected into the SN). A previous study testing various titers of AAV-GFP and AAV-α-Syn demonstrated that, at a titer of 2 x 10^14^ gc/mL and higher, AAV-GFP resulted in approximately a 62% loss of TH-positive neurons in the substantia nigra [1]. The amount of AAV viral particles used in the current study is within the safe range [2, 3]. However, as suggested, we compared the number of TH-positive neurons between the contralateral and ipsilateral sides, showing no significant difference with a slightly less number in the ipsilateral SN (t = 1.787, *p* = 0.104). We have added this result as S2 Fig.

[1] Landeck, N., Buck, K., & Kirik, D. (2017). Toxic effects of human and rodent variants of alpha‐synuclein in vivo. European Journal of Neuroscience, 45(4), 536-547.

[2] Klein, R. L., Dayton, R. D., Leidenheimer, N. J., Jansen, K., Golde, T. E., & Zweig, R. M. (2006). Efficient neuronal gene transfer with AAV8 leads to neurotoxic levels of tau or green fluorescent proteins. *Molecular therapy*, *13*(3), 517-527.

[3] Koprich, J. B., Johnston, T. H., Huot, P., Reyes, M. G., Espinosa, M., & Brotchie, J. M. (2011). Progressive neurodegeneration or endogenous compensation in an animal model of Parkinson's disease produced by decreasing doses of alpha-synuclein. *PloS one*, *6*(3), e17698.

S2 Fig.

**Q8**. Page 5-6: How does the saline correlate with NXP031 solvent? Was NXP031 also dissolved to saline in the end?

**A8**. We prepared Aptamin C stock (1mg/ml) and diluted it with the saline. Then, an appropriate dose of vitamin C for oral gavage was dissolved. We have added it to the methods section.

NXP031 preparation

Development and characterization of Aptamin C has been previously described [27]. The purified DNA aptamer (Aptamin C) was obtained from Integrated DNA Technologies (IDT, IA, USA). Aptamin C was dissolved in folding buffer (1 mM MgCl2 in 0.01 M PBS), heated in boiling water at 90 – 95 ℃ for 5 min, and cooled slowly at room temperature to fold into a tertiary structure. Aptamin C stock (1mg/ml) was prepared and diluted with saline to adjust the concentration of 4mg/kg for oral gavage. NXP031 was prepared by freshly mixing L-ascorbic acid (ThermoFisher Scientific, MA, USA) with a Aptamin C in a ratio of 1:50 (w/w) before oral gavage.

**Q9**. Page 8: TH+ neuron count. More details are needed. Was stereology taken into account as the authors used 30 um sections?

**A9**. We selected every sixth section from the beginning of the SN to cover the entire SN area. After TH staining in total 6 tissues for each animal, stereological counting was performed. We have added the quantitative analysis method of TH immunohistochemistry with stereological counting. According to the reviewer's opinion, this content has been added to Materials and Methods.

Unbiased stereological counting

For unbiased stereological counting, we estimated the number of TH-positive neurons in the SN using Leica DM4B motorized microscope equipped with StereoInvestigator software (MicroBrightField Bioscience, Williston, VT, USA). The total number of TH-positive neurons was estimated according to the optical fractionator workflow probe. Every sixth section (30 µm thickness), from the anterior to the posterior midbrain, was analyzed. The ipsilateral SN was first outlined at low magnification (5x). The counting frame size was set 60 x 60 µm, the grid frame size was set 120 x 120 µm. Top and bottom guard zones were applied to each site, and an optical dissector height of 25 µm was used. Counts for TH-positive neurons were performed at high magnification (20x). An acceptable cell estimation had a coefficient of error (CE) using the Gunderson method (m=1) less than 0.1.

Results:

**Q10**. Figure 4. The authors state that they didn’t detect 4-HNE staining in SNPc but in the Figure 4, there is clearly some staining visible. Why this was not analyzed or did the authors conclude that this is not specific?

**A10**. As carefully explained in the beginning part of this response letter, severe DA neuronal loss in the AAV-WT-α-Syn group makes it difficult to assess 4-HNE. Although there were some remaining neurons express higher levels of 4-HNE, it is not reliable to compare them with the AAV-WT-α-Syn + NXP031 group.

Discussion:

**Q11**. TH loss does not always refer to loss of DAergic neurons, and this is also visible in Suppelementary Figure S1. This should be taken into account in the discussion.

**A11.** Thank you for providing these insights. We have added this to the discussion section.

Discussion

At 9 weeks after the human WT-α-Syn/AAV-injection into the SN, we assessed the motor function and dopaminergic neuronal survival at the injection site. Consistent with previous studies, AAV-mediated α-Syn overexpression in the SN of mice led to a significant reduction of TH-positive neurons, leading to motor dysfunction [38-40]. To make sure dopaminergic neuronal loss, we also used Nissl staining, indicating a loss of both TH and Nissl staining as dopaminergic neuronal degeneration. NXP031 treatments ameliorated motor deficits and dopaminergic neuronal death in the SN. These results support our previous study that NXP031 protects acute degeneration of nigral dopaminergic neurons in the MPTP-induced mitochondrial oxidative stress PD model [21] and expand its application to α-synucleinopathy-based PD models.

**Reviewer#2**. Song et al report that 1 week following unilateral infusion of AAV-a-syn into the SN, daily oral administration of NXP031 for the next 8 weeks is neuroprotective in terms of blocking the alpha synuclein-induced motor dysfunction as determined using the rotorod, and blocking the alpha synuclein-induced loss of TH neurons in the SNpc. Although there was no evidence that NXP blocked the aggregation of alpha synuclein within the SNpc, since the aggregation was apparently located in non-neuronal cells, the authors quantified the aggregation within the hippocampus. This alpha synuclein-induced aggregation was blocked by prior administration of NXP within both the DG and CA3. Only within the hippocampus did NXP block the accumulation of 4-HNE, a marker of oxidative stress, and H2A.X (histone phosphorylation). Since this is a neuroprotective study, it is more appropriate to use the term, blocked, versus ameliorated. Although the aggregation extended from the SN to the hippocampus, the authors provide no behavioral data to suggest this aggregation was deleterious, which is a major limitation of this study. Also, NXP blocked the aggregation of alpha synuclein and did not reduce the aggregation. 'Reducing' implies that there was previous aggregation but this is incorrect, since this was a neuroprotective study design. In addition, since here are no prodromal biomarkers that can diagnose PD years prior to the emergence of the motor dysfunction, a neuroprotective study design is not translational to the clinic at this time. Therefore, the authors have over-interpreted their findings that NXP is a potential therapeutic for PD.

**Q1**. Abstract: based on the comments above, the authors need to change the wording of the abstract (ie., reduced) and decrease the emphasis of the last sentence.

**A1**. We genuinely appreciate all comments of reviewer #2. We have changed the abstract.

Abstract

Parkinson’s disease (PD) is a neurodegenerative disease characterized by inclusions of aggregated α-synuclein (α-Syn). Oxidative stress plays a critical role in nigrostriatal degeneration and is responsible for α-Syn aggregation in PD. Vitamin C or ascorbic acid acts as an effective antioxidant to prevent free radical damage. However, vitamin C is easily oxidized and often loses its physiological activity, limiting its therapeutic potential. The objective of this study was to evaluate whether NXP031, a new compound we developed consisting of Aptamin C and Vitamin C, is neuroprotective against α-synucleinopathy. To model α-Syn induced PD, we stereotactically injected AAV particles overexpressing human α-Syn into the substantia nigra (SN) of mice. One week after AAV injection, NXP031 was administered via oral gavage every day for eight weeks. We found that oral administration of NXP031 ameliorated motor deficits measured by the rotarod test and prevented the loss of nigral dopaminergic neurons caused by WT-α-Syn overexpression in the SN. We also observed that NXP031 blocked the propagation of α-Syn pathology into the hippocampus by alleviating oxidative stress. These results indicate that NXP031 can be a potential therapeutic for PD.

**Q2**. Materials: Animals: the authors need to include an NXP only treated group that was originally infused with the AAV-empty vector group to determine if the drug has its own effects. Therefore, the 3 groups analyzed in this study is incomplete. Was the saline also administered orally? This should be included.

**A2-1**. We agree with the reviewer’s comments. We did not include the AAV-GFP + NXP031 group because, as expected, the initial TH staining of this group did not show a difference from the AAV-GFP group. We did not include this group in the original manuscript to reduce time and effort. According to the reviewer’s advice, we included the AAV-GFP + NXP031 group in the revised manuscript. We rewrote the methods and the results sections accordingly.

**A2-2**. Both the AAV-GFP and the AAV-WT-α-Syn groups were orally administered with saline. We have revised the Materials and Methods’ experimental designs section to clarify this.

Experimental designs

Recombinant AAV vectors were produced by modification of pAAV-IRES-hrGFP (Agilent) to express wild-type human α-Syn under control of the CMV promoter. Viral particles (AAV2) were produced at the University of Iowa Viral Vector Core according to their standard operating procedures (https://medicine.uiowa.edu/vectorcore/). For viral injection, the mice were deeply anesthetized with 3% isoflurane and placed in a stereotactic instrument (Stoelting, IL, USA). After making a midline incision of the scalp and a burr hole in the skull over the appropriate injection site for the SN, all mice were stereotactically injected unilaterally into the right SN with a microinjector at a rate of 0.25 μl/min with either 2 μl of GFP-only AAV or 2 μl of WT-α-Syn AAV at a concentration of 2 x 1012 viral genomes/mL. The needle was left in place for an additional 8 min before it was slowly withdrawn. Stereotaxic coordinates for the SN were as follows: anteroposterior (AP) -3.0 mm, mediolateral (ML) -1.4 mm, and dorsoventral (DV) -4.4 mm from the skull surface. One week following AAV injection, NXP031 (Vitamin C/Aptamin C 200 mg/4 mg/kg, dosage determined in previous studies [18]) was administered to the α-Syn-NXP031 group via oral gavage every day for 8 weeks. The AAV-GFP and the AAV-WT-α-Syn group groups were orally administered with saline. 8 weeks after NXP031 or saline treatment, rotarod test was carried out. All mice were deeply anesthetized with 3% isofluorane and were sacrificed by transcardial perfusion with saline followed by 4% PFA (Fig 1A).

**Q3**. Experimental design: The authors need to demonstrate how far the AAV-alpha synuclein spread throughout the SN. Using the GFP-only AAV, this needs to be carried out. The concern of this reviewer is that the authors needed to remove a group of animals 1 week after the AAV-a-syn infusion, and prior to the start of the NXP treatment, to determine if there was any loss of TH labeled neurons in the SNpc. This group needs to be included.

**A3**. We agree with the reviewer’s comments. In our pilot experiment, no TH+ dopaminergic neuronal loss was observed at 1 week after WT α-Syn/AAV injection into the SN (Fig A, below). 1 week after injection, GFP expression was limited at the injection site, but it gradually spreads to the entire SN after 9 weeks (Fig B, below). We added Fig A (below) in Fig. S1.

**Q4**. Rotorod test: when did the animals undergo the 3 days of training sessions? Assume prior to the start of the AAV-infusion? Please clarify.

**A4**. We have revised the sentence.

Rotarod test

The rotarod test is widely use to assess balance and motor coordination of rodents as previously described [18]. A rotarod machine (Ugo Basile, Coerio, Italy) was used to record the latency time to fall on the rotating rod. All mice had 2 days of training before the start of measurement, where each mouse was placed upon the rotor at a low speed of 10 rpm for 60 s. The next day, mice were tested at automatically accelerated speed from 0 to 30 rpm. When the mice fell off the rod, the latency time to fall was recorded for 480 s.

**Q5**. Immunohistochemistry: although it was later explained that following the infusion of AAV-a-syn, the protein is distributed/transported to various regions, including the hippocampus, a clear experimental design flaw in this study was that there were no behavioral experiments to determine if the aggregation, increase in ROS and double stranded breaks in the hippocampus lead to a deficit in learning/memory. Considering the authors carried out rotarod testing, was there aggregation within the motor cortex? The hippocampal data, although of interest, has little to do with the neuroprotective effects in the SNpc. For the SNpc/TH analysis, was the entire rostral-caudal extent of the SN sectioned and analyzed? This is important since it's not clear how far the AAV infusion spread along the SN and whether the infusion spread outside the SN. For the hippocampus, how many sections were analyzed?

**A5-1**. As carefully explained in the beginning part of this response letter, we think α-Syn pathology, DNA damage, and oxidative stress observed in the hippocampus were used as indicators for strong α-Syn pathology that starts in the SN. Therefore, our study did not focus much on cognitive functional recovery by this therapy. Changes in motor behavior assessed by rotarod test were mainly intended to gauge nigrostriatal dopaminergic system.

**A5-2**. For analysis of TH-positive neurons in the SN, we selected every sixth section from the beginning of the SN to cover the entire SN area.

**A5-3**. We used 2 sections per animal for hippocampus analysis. Also, we have added this information to the methods section.

**Q6**. TH-positive cells counting: please provide more detail as to how the cell count was carried out. Was this done stereologically?

**A6**. We selected every sixth section from the beginning of the SN to cover the entire SN area. After TH staining in total 6 tissues for each animal, stereological counting was performed. We have added the quantitative analysis method of TH immunohistochemistry with stereological counting. According to the reviewer's opinion, this content has been added to Materials and Methods.

Unbiased stereological counting

For unbiased stereological counting, we estimated the number of TH-positive neurons in the SN using Leica DM4B motorized microscope equipped with StereoInvestigator software (MicroBrightField Bioscience, Williston, VT, USA). The total number of TH-positive neurons was estimated according to the optical fractionator workflow probe. Every sixth section (30 µm thickness), from the anterior to the posterior midbrain, was analyzed. The ipsilateral SN was first outlined at low magnification (5x). The counting frame size was set 60 x 60 µm, the grid frame size was set 120 x 120 µm. Top and bottom guard zones were applied to each site, and an optical dissector height of 25 µm was used. Counts for TH-positive neurons were performed at high magnification (20x). An acceptable cell estimation had a coefficient of error (CE) using the Gunderson method (m=1) less than 0.1.

**Q7**. Statistical analysis: please include the Degrees of Freedom in your ANOVA. In addition, with the needed inclusion of a 4th experimental group (VEH+NXP), the authors will need to carry out a 2-way ANOVA.

**A7.** We included the 4th experimental group (AAV-GFP + saline group) and changed the statistical method to 2-way ANOVA. The degrees of freedom in the results of 2-way ANOVA was included as suggested. The results and legend were carefully re-written accordingly.

Statistical analysis

Statistical analyses were performed using GraphPad Prism 9.3.1 (GraphPad Software Inc., CA, USA). Two-way analysis of variance (ANOVA) was used in comparing means of all the groups, followed by Tukey’s post-hoc tests for multiple comparisons. All data were presented as means ± S.E.M. The criterion for significance was set at *p* < 0.05.

**Q8**. Where is the Figure Legend for Figure #1? Please include.

**A8.** The legend of Fig 1 was on page 6 in Materials and Methods of our manuscript as we follow the manuscript preparation guidance.

Fig 1. (A) Experimental paradigm. (B) Effect of NXP031 on the rotarod test at 9 weeks after delivery of GFP-only or α-Syn AAV in the SN. Latency to fall time (sec) in the rotarod test. Data are presented as the mean ± S.E.M. (Two-way ANOVA followed by Tukey’s test, **p* < 0.05, ****p* < 0.001 compared to selected group, n= 6-8 mice per group).

**Q9**. Line 191: were there rotorod motor difficulties 1 week following the infusion of AAV-a-syn but prior to the start of the NXP treatment? That would be important data to know.

**A9.** We did not perform the rotarod test at 1 week after the AAV-WT-a-Syn injection into the SN. However, as mentioned in Q3, loss of TH-positive neurons was not observed 1 week after AAV-WT-α-Syn injection, suggesting no motor deficit at this time point.

**Q10**. Line 200: similar to the comment in #9 above, how much loss of TH neurons were there 1 week after AAV-a-syn infusion but prior to the start of NXP treatment. Also please change the heading of that section to 'NXP blocks ....'.

**A10.** As mentioned in Q3, our pilot study showed no TH+ dopaminergic neuronal loss 1 week after AAV-WT-a-Syn injection. We have changed the heading of that results to “NXP031 blocks the loss of dopaminergic neurons against AAV-mediated overexpression of human α-Syn in the SN”.

**Q11**. Line 206: in the S1 Figure, since these are double stained sections for TH and CV, the authors need to quantify the number of TH negative/CV positive neurons and not just show the photos. In the S1 figure, far right photographs, it appears there is loss of TH+ cells on the contralateral (left) side of the SNpc. The authors now need to also quantify the number of TH+ neurons on the contralateral side since it appears that there was possibly spread of the AAV-a-syn to the contralateral SN. In this S1 figure, the authors need to show a photo of the vehicle only control.

**A11-1.** AAV-mediated unilateral overexpression of α-Syn can spread to the contralateral side over time. As the reviewer noted, we observed a slight loss of TH+ neurons in the contralateral SN. This was expected and reported in this model. As we did not expect that contralateral TH+ neuronal counting does not affect the overall outcome of the study, we did not include it.

**A11-2**. According to the reviewer’s suggestion, we changed the representative images in the S1 Figure and moved them to Fig 2F. We wanted to focus on dopaminergic neuronal loss in the results of Nissl staining, so we quantified the percentage of Nissl stained neurons (% of AAV-GFP) and added it to Fig 2G.

Fig 2.

**Q12**. Line 224: the authors need to double label for p-129 and TH to determine if the p-129 is located in non-dopamine neurons.

**A12**. As suggested, we performed double immunostaining of TH and pS129-α-Syn, demonstrating that pS129-α-Syn staining is mostly co-stained with TH+ neurons. This new result was added to Fig 3A.

Fig 3.

**Q13**. Line 230: as mentioned before, the lack of any learning/memory correlate to the increased aggregation in the DG/CA3 limits the interpretation of this finding and has little to do with the main theme of this study, which was to determine the effects of the NXP treatment in terms of PD.

**A13**. The answer to this question was carefully explained in A5-1.

**Q14**. Line 239: Because no learning/memory behavioral studies were carried out, the authors cannot say that there was no toxicity associated with the aggregation. Toxicity does not necessarily imply cell loss. Aggregation within neurons in the DG/CA3 may have affected the function of the cells, which some would interpret as a toxic effect. This line needs to be changed or eliminated. The same goes for line 321 in the discussion section. This line needs to be changed or eliminated.

**A14**. We have revised these sentences.

Results

The result suggests that the hippocampal regions are relatively resistant to α-Syn-mediated cell death, and NXP031 can effectively prevent the propagation of α-Syn into the hippocampus.

Discussion

To circumvent this problem, we investigated other brain regions relatively resistant to α-Syn –mediated cell death

**Q15**. Figure 3A: In the far right photo showing the effects of alpha synuclein, how do the authors know this is the SNpc? The authors need to show a much higher magnification. Since this reviewer is a basal ganglia morphologist, it's difficult to determine the exact location of the labeled cells. They actually appear to be dorsal to the SNpc. In addition, what cell type is labeled with p-129 in these photos?

**A15**. The TH staining image taken at 2.5x attached below shows that TH staining is specific to dopaminergic neurons, and we could distinguish the SNpc region. We added a photo taken at 2.5x to show the location of the SNpc in Fig 2. We also included the result of double immunostaining of TH and pS129-α-Syn in Fig 3, showing that pS129-α-Syn was co-stained with TH.

**Q16**. Line 250: need to change, suppresses, to 'blocks'. Also in Figure 4, there was no 4-HNE staining of the SN, it appears that the loss of TH cells following AAV-a-syn has nothing to do with oxidative stress or DNA double breaks. Please comment in the discussion section.

**A16-1**. We have changed the heading of that results to “NXP031 blocks oxidative stress and DNA double-strand breaks”.

**A16-2**. We have changed the representative images of the 4-HNE staining in the SN. We elaborated explanation as to the association between oxidative stress or DNA double breaks and dopaminergic neuronal loss in Discussion.

**Discussion**

Oxidative stress is one of the main factors contributing to dopaminergic neuronal degeneration in PD, inducing oxidative damage to proteins, lipids, and DNA [43, 44]. We then showed that NXP031 blocked oxidative stress induced by α-Syn overexpression in the SN. 4-HNE staining for toxic products of oxidative damage is a widely used biomarker for increased ROS levels, and we found that overexpression of α-Syn led to increased levels of 4-HNE staining. A previous study has indicated that 4-HNE-modified proteins were significantly increased in nigral melanized neurons in human post-mortem brain PD samples [45]. The AAV-mediated A53T-α-Syn overexpression in the SN of rats has shown a significant increase in 4-HNE staining [39]. Our results are consistent with the previous studies [39, 45], suggesting that α-Syn pathology-mediated oxidative stress may contribute to nigral neuronal cell death. Similar to pS129-α-Syn staining, severe dopaminergic neuronal loss caused by α-Syn overexpression made it challenging to detect 4-HNE in the SN. Alternatively, we found a significant increase in 4-HNE levels accompanied by pS129-α-Syn staining in the hippocampus, implying the propagation of α-Syn pathology from the SN. We thought that NXP031 treatments prevented α-Syn-mediated oxidative stress and DNA damage primarily in the SN and further blocked its propagation into the hippocampus.

DNA double-strand break is the most harmful DNA damage, which triggers activation of phosphorylation of the histone variant H2A.X, a significant component of DNA damage response [36]. Increased DNA damage markers such as γH2A.X, 53BP1, and pATM have been reported in two different synucleinopathy models of PD, demonstrating that α-Syn-mediated oxidative stress results in DNA double-strand breaks [46]. We similarly observed both γH2A.X and 4-HNE staining were elevated in the SN and hippocampus. While PD has strong connections with reactive oxygen species, other neurodegenerative conditions such as Alzheimer’s disease and multiple ALS have also been shown to have oxidative damage components and it will be worth it to investigate the potential of this compound on those conditions in future studies.

Fig 4.

**Q17**. In Figure 4 and Figure, how many hippocampal sections were taken for the cell counts? Did these sections cover the entire rostral-caudal extent of both the DG and CA3? This information should be included in the methods section. For Figure 5, where is the SN data?

**A17-1**. We used 2 sections per animal for hippocampus analysis. Unlike SN, for the hippocampus, the cell distribution pattern was constant along the rostral to caudal, so we thought that 2 sections were sufficient. We added this to the methods section.

**A17-2**. We have added the representative image of pH2AX staining in the SN into Fig 5A.

Fig 5.

**Q18**. Discussion section: Page 16: As noted in comment #16 above, the authors need to provide an explanation as to their hypothesis as to how the dopamine cells are dying since it does not appear to be associated with either increased ROS or DNA double strand breaks. This is why a group of mice should have been removed from the study at the end of the first week following AAV-a-syn infusion, since it's possible the remaining SNpc neurons might have labeled for both markers. So how does NXP block the a-syn induced loss of DA/TH cells if not via blocking ROS or DNA double strand breaks? The authors should have carried out TH IHC in the striatum since it's highly possible that there might have been sprouting of new DA terminals in the striatum due to NXP treatment.

**A18-1**. As carefully explained in the beginning part of this response letter and A5-1, evident neuronal loss prevents us from seeing changes in ROS and DNA double-strand break in the SN. However, reductions in those pathologic markers in the hippocampus by NXP031 indirectly imply that dopaminergic neuronal protection in the SN could also be attributed to the same mechanisms. To address this invaluable question, it might be necessary to conduct a more careful time-course experiment to validate this α-Syn overexpression model system further.

**A18-2.** As suggested, we added TH-staining in the striatum, showing the protection of dopaminergic neuron fibers in Fig. 2A and B.

Fig 2.

**Q19**. Conclusion: the authors need to note the blocking of the aggregation was in the hippocampus and not the SNpc and that they need to tone down the idea that is drug could be used as a therapeutic since this was a neuroprotective study design.

**A19**. The answer for this question was discussed several times above. We revised explanation in Discussion as suggested.

Discussion

In conclusion, our results demonstrate the neuroprotective promise of NXP031 in the AAV-WT-α-Syn mouse model of PD by preventing dopaminergic neuronal loss in the SN and inhibiting the propagation of α-Syn pathology further into the other brain regions through reducing oxidative stress. Thus, we suggest that NXP031 could represent a prospective therapeutic strategy for PD.
