# Supplementary material for: NXP031 prevents dopaminergic neuronal loss and oxidative damage in the AAV-WT-α-synuclein mouse model of Parkinson’s disease
Source: PLoS One. 2022 Jul 28;17(7):e0272085. doi: 10.1371/journal.pone.0272085 (PMC9333296; doi:10.1371/journal.pone.0272085)
Supplement: Attachment — Submitted filename: Response to reviewers 2nd.docx [file pone.0272085.s004.docx]

Hemant K. Paudel

Academic Editor

PLOS ONE

We appreciate the constructive comments made by reviewers and for giving us the opportunity for revision. We have revisited the entire manuscript and thoroughly revised the manuscript accordingly, and we are confident that our manuscript satisfies reviewers’ concerns and is greatly improved. We performed grammar corrections mentioned by the reviewer and carefully conducted the reference check journal requirements.

Reviewer's Comments to Author

**Reviewer#2**. Song et al have significantly revised their manuscript but this reviewer has a few more concerns that need to be addressed:

**Q1**. Supplemental Figure 1: this reviewer found it of interest that following AAV-a-syn infusion into the SNpc, that only about 50% of the SNpc neurons were GFP labeled. I am assuming the 50% loss of TH cells after 9 weeks in the SNpc following alpha synuclein infusion were only those cells containing the alpha synuclein? Since the TH stereology was carried out throughout the entire rostral-caudal extent of the SNpc, the authors need to determine the percentage of SNpc neurons containing the alpha synuclein after just one week (since it was shown that there was essentially no loss of TH neurons at this early time period). Was the alpha synuclein induced loss of TH cells uniform throughout the entire SNpc or, since there is most likely complete loss of TH in the immediate area of the viral infusion, as essentially stated by the authors, was there significantly less loss further away from the injection site? These data are important in terms of interpreting the protective effects of the NXP031 drug administered. Since there was about a 50% loss of TH cells in this study, and if there was nearly 100% loss at the site of the infusion, this suggests that further away from the injection site, there must have been nearly no loss of TH cells. This needs to be clarified.

**A1**. As we mentioned in the previous revision, 1 week after injection, GFP expression was limited at the injection site, but it gradually appeared in the entire substantia nigra (SN) at 9 weeks (Under Fig A). This is reasonable because it is expected that a viral titer should be highest at the immediate injection site with gradual decreases in the surrounding areas. In our injection protocol, however, we tried to inject AAV particles very slowly at 0.25 ul/min over 8 minutes and wait another 5 min before removing a needle to evenly spread viral particles as much as possible. Below in fig B, we presented the full TH immunostaining images obtained by performing TH immunostaining on every 6th SN tissue 9 weeks after injection. The loss of TH-positive cells after 9 weeks was not limited to the injection site, and it was observed uniformly throughout the entire SN. We think that 9 weeks after injection of AAV-WT-αSyn viral particles, the α-Synuclein (α-Syn) expression reached the threshold in the whole rostral-caudal SN to induce dopaminergic neuronal death throughout the SN.

**Q2**. Line 139: the authors state the animals were trained for 2 days before the start of the measurement. This reviewer assumes the mice were trained after the 9 weeks of AAV infusion? If that is the case, please state it more specifically. Initially the authors had stated the mice were trained for 3 days. Which is correct? Please clarify.

**A2**. The original manuscript described “All mice had 3 training sessions prior to measurement” and the revised manuscript stated “the animals were trained for 2 days before the start of the measurement”. All the methods we mentioned are correct. We have revised the rotarod method section as below.

Rotarod test

The rotarod test is widely used to assess the balance and motor coordination of rodents, as previously described [21]. A rotarod machine (Ugo Basile, Coerio, Italy) was used to record the latency time to fall on the rotating rod. All mice were trained on the rotarod for 2 consecutive days at a fixed speed of 10 rpm for 60 s. Mice were received 3 training trials per day with a 1 h inter-trial interval. The next day, mice were tested at an automatically accelerated speed from 0 to 30 rpm. When the mice fell off the rod, the latency time to fall was recorded, up to a maximum of 480 s.

**Q3**. Figure 2F/G: the legend states (line 244/245) the following: Representative micrographs of double staining with TH antibody and Nissl violet in the SN. (G) % of Nissl-positive neurons compared to the AAV GFP group. For this analysis, this reviewer assumes that the authors counted both TH positive and Nissl only positive cells, or were just the Nissl positive and TH NEGATIVE cells counted? In the original question asked by this reviewer, Point #11, only the Nissl positive and TH NEGATIVE cells should be counted in order to determine if there was a change in those numbers. The reason for this request is that this author has reported several times that following loss of nigrostriatal TH cells, there was an increase in the number of TH negative/Nissl positive cells in the SNpc, suggesting that some of the original TH positive cells had not died but simply were not expressing the TH protein at the moment. This reviewer has interpreted the data from Figure 2G as the authors counting all TH positive/Nissl stained cells. Please clarify.

**A3**. In the SN, there are other types of neurons in addition to TH-positive dopaminergic neurons, making the number of Nissl-positive neurons consistently higher than TH-positive neurons. If only the number of TH-positive cells is reduced without accompanying reduction in Nissl staining, it may be due to transient changes in TH expression levels without actual dopaminergic neuronal death, as the reviewer stated. Therefore, similar reductions in both TH and Nissl staining are only considered as a dopaminergic neuronal loss. We counted TH- or Nissl-positive cells separately and observed a similar reduction that was recovered by drug administration. This counting method has been widely used and well appreciated for the reliable measurement of dopaminergic neuronal death. This observation clearly suggests αSyn/AAV-mediated dopaminergic neuronal loss was blocked by NXP031. We described more clearly in the result as below.

NXP031 blocks the loss of dopaminergic neurons against AAV-mediated overexpression of human α-Syn in the SN.

To confirm that the decreased TH immunoreactivity was caused by neuronal loss and not by transient decreases in TH expression changes, we performed Nissl staining (Fig 2F and G). We observed similar reduction in the number of Nissl-positive cells in the SN at 9 weeks after α-Syn overexpression. Together with decreased TH-positive staining, this result indicates dopaminergic neuronal degeneration (AAV vector type x treatment interaction F_1, 24_ = 8.187, *p* = 0.009). The result implies that NXP031 efficiently blocked dopaminergic neuronal death caused by α-Syn overexpression in the SN.

**Q4**. This author is still not convinced as to why the hippocampal data are included without any behavioral testing. The fact that there was no hippocampal cell loss, but there was still significant aggregation/increased p-alpha synuclein labeling, suggests to this reviewer that learning/memory deficits are most likely present. Of interest, there are recent data suggesting that during the training of a learning/memory task, there is an actual increase in DSBs in the hippocampus, suggesting that such breaks and increased repair are essential to the learning of certain tasks. Therefore, the testing of these alpha synuclein-infused mice in a L&M task is even more important. Why didn't the authors investigate p-alpha synuclein labeling in the striatum? This reviewer has found, using essentially the same AAV-alpha synuclein viral infusion into the SN, a clear increase in p-alpha synuclein in the striatum. Since the SNpc projects to the striatum, as it appears to also do to the hippocampus, it would have made more sense to investigate the striatum. Please comment.

**A4-1**. The answer to this question was extensively explained in the previous responses. The primary aim of using this model is to test if NXP031 effectively reduces or blocks α-Syn aggregation-induced dopaminergic (DA) neuronal loss in the SN (**not hippocampal dysfunction or loss**). To assess the therapeutic efficacy, we measured 1) the number of TH-positive neurons in the SN, 2) pS129-α-Syn as a marker for α-Syn aggregation, 3) 4-HNE as oxidative lipid peroxidation, and 4) γH2A.X as a DNA double-strand break. DA neuronal loss in the saline ingested group was significantly obvious compared to the NXP031 treated group. **This evident DA neuronal loss invited difficulty in measuring other pathology markers (2-4) as all markers express inside cells and disappear with degeneration**. Therefore, we investigate the hippocampus as an alternative region for two reasons: 1) propagation of α-Syn aggregates from the SN to the hippocampus over time is well documented (ref below), 2) hippocampal neuronal loss is minimal even though α-Syn pathology is observed. This is why we did not pay much attention to functional changes in the hippocampus. Together with TH-positive neuronal count and rotarod test, this result suggests that NXP031 blocked αSyn-induced dopaminergic neuronal degeneration in the SN.

Kim, S., Kwon, S. H., Kam, T. I., Panicker, N., Karuppagounder, S. S., Lee, S., ... & Ko, H. S. (2019). Transneuronal propagation of pathologic α-synuclein from the gut to the brain models Parkinson’s disease. *Neuron*, 103(4), 627-641.

**A4-2**. Although we do not know exactly what kind of paper the reviewer found, we also found a recent paper that confirmed α-Syn aggregation in the striatum in the AAV α-Syn model of PD (ref below). A recent paper showed the expression of pS129 in the striatum by WB 10-11 weeks after AAV-WT-αSyn injection into the SN. Compared to the contralateral site, the expression of pS129 in the ipsilateral site was not statistically different (fig below). We checked whether α-Syn aggregation was expressed in our PD model's striatum, but pS129 staining was not observed in the striatum.

Fig. S3C. Representative western blot showing immunoreactivity of pSer129-α-synuclein and tyrosine hydroxylase (TH) in protein lysates from the striatum. Fig. S4B. The immunoreactivity signal for pSer129-α-synuclein has been standardized to STEP46, and then a ratio of left to right has been computed. An unpaired t-test with inequal variance showed t(14) = -1.566, *p* = 0.16. (Reference: Østergaard, F. G., Himmelberg, M. M., Laursen, B., Siebner, H. R., Wade, A. R., & Christensen, K. V. (2020). Classification of α-synuclein-induced changes in the AAV α-synuclein rat model of Parkinson’s disease using electrophysiological measurements of visual processing. *Scientific reports*, *10*(1), 1-14).

**Q5**. There are still some minor grammar issues that need to be corrected.

**A5**. According to the reviewer’s advice, the revised manuscript with a careful English editing service.
